# Artificial intelligence in language instruction: impact on English learning achievement, L2 motivation, and self-regulated learning

**DOI:** 10.3389/fpsyg.2023.1261955

**Published:** 2023-11-06

**Authors:** Ling Wei

**Affiliations:** College of Foreign Languages, Chongqing College of Mobile Communication, Chongqing, China

**Keywords:** AI-mediated language instruction, English learning achievement, L2 motivation, self-regulated learning, EFL learners, mixed-methods approach

## Abstract

**Introduction:**

This mixed methods study examines the effects of AI-mediated language instruction on English learning achievement, L2 motivation, and self-regulated learning among English as a Foreign Language (EFL) learners. It addresses the increasing interest in AI-driven educational technologies and their potential to revolutionize language instruction.

**Methods:**

Two intact classes, consisting of a total of 60 university students, participated in this study. The experimental group received AI-mediated instruction, while the control group received traditional language instruction. Pre-tests and post-tests were administered to evaluate English learning achievement across various domains, including grammar, vocabulary, reading comprehension, and writing skills. Additionally, self-report questionnaires were employed to assess L2 motivation and self-regulated learning.

**Results:**

Quantitative analysis revealed that the experimental group achieved significantly higher English learning outcomes in all assessed areas compared to the control group. Furthermore, they exhibited greater L2 motivation and more extensive utilization of self-regulated learning strategies. These results suggest that AI-mediated instruction positively impacts English learning achievement, L2 motivation, and self-regulated learning.

**Discussion:**

Qualitative analysis of semi-structured interviews with 14 students from the experimental group shed light on the transformative effects of the AI platform. It was found to enhance engagement and offer personalized learning experiences, ultimately boosting motivation and fostering self-regulated learning. These findings emphasize the potential of AI-mediated language instruction to improve language learning outcomes, motivate learners, and promote autonomy.

**Conclusion:**

This study contributes to evidence-based language pedagogy, offering valuable insights to educators and researchers interested in incorporating AI-powered platforms into language classrooms. The results support the notion that AI-mediated language instruction holds promise in revolutionizing language learning, and it highlights the positive impact of AI-driven educational technologies in the realm of language education.

## 1. Introduction

The application of information technology in language learning and teaching has garnered considerable attention from language researchers in recent years (Ahmadi, 2018; Shadiev and Yang, 2020; Lai et al., 2022; Lei et al., 2022; Soleimani et al., 2022; Shadiev et al., 2023). Leveraging information technology in language education can enhance the learning experience for learners by enabling personalized, interactive, and communicative learning processes (Rodinadze and Zarbazoia, 2012; Chun et al., 2016; Shatri, 2020; Rahimi and Fathi, 2022). Language educators have adopted information technology to create virtual language learning environments that actively engage learners and facilitate language learning processes (Fathi and Rahimi, 2022; Loncar et al., 2023; Nguyen and Le, 2023). Among the information technology programs, artificial intelligence (AI) has emerged as a promising tool applied in language learning and teaching to enhance learners’ learning achievements (Haristiani, 2019; Pedro et al., 2019; Knox, 2020; Pikhart, 2020; Huang et al., 2023).

In the context of computer programs, AI is purposefully designed to interpret and respond to human queries, operating as a platform that relies on human intelligence to furnish pertinent information (Buzko et al., 2016; Spector and Ma, 2019; Devi et al., 2022; Khosravi et al., 2022; Nemorin et al., 2023). For example, ChatGPT, an AI-equipped tool, can efficiently supply users with requested information based on their queries (Fitria, 2023; Yan, 2023). In fact, the emergence of AI has ushered in transformative changes across various industries, and the field of education and language learning is no exception (Spiro et al., 2017; Balyen and Peto, 2019; Su et al., 2023). AI’s potential to revolutionize traditional teaching and learning methods has captured the attention of educators, researchers, and policymakers worldwide (Michalski et al., 2013; Ilkka, 2018). With its ability to process vast amounts of data, analyze complex patterns, and deliver personalized insights, AI offers new possibilities for enhancing educational practices and student outcomes (e.g., Roll and Wylie, 2016; Ouyang and Jiao, 2021). Within this direction, educators have integrated AI-assisted language learning tools into education to support learners in enhancing their language skills (Lu, 2018; Tafazoli et al., 2019). ChatGPT, as an AI-assisted language learning tool, offers potential benefits for language learners’ language skills and subskills (Baskara and Mukarto, 2023; Hong, 2023; Kohnke et al., 2023). It provides learners with writing ideas, suggests alternative sentences to improve their writing performance, and contributes to their language learning achievements (Su et al., 2023; Yan, 2023). AI-supported language learning tools are known for creating immersive and engaging learning environments, allowing learners to conveniently undertake language learning tasks and improve their overall language proficiency (Divekar et al., 2022).

Several research studies have explored the influence of AI-assisted language learning tools on English language learners’ overall learning achievement and specific language skills and sub-skills (Kim, 2019; Junaidi, 2020; Zheng et al., 2021; Xu et al., 2022; Hsu et al., 2023; Yan, 2023). For instance, Xu et al. (2022) conducted a study investigating the impact of AI-powered language learning tools on English language learners’ overall learning achievement and found a positive contribution of AI-assisted language learning tools to learners’ achievement. Another study by Hsu et al. (2023) examined the effects of AI-assisted language learning tools on EFL learners’ vocabulary knowledge and revealed that learners utilizing AI tools demonstrated significant improvement and outperformed their peers in vocabulary knowledge. Moreover, Junaidi (2020) investigated the role of AI-assisted language learning tools in enhancing EFL learners’ speaking skills and found that AI learners outperformed non-AI learners in speaking proficiency.

Despite these valuable insights, prior research has not sufficiently delved into the impact of AI-supported instruction on English language learners’ language learning achievement, particularly in the context of English as a foreign language (EFL). Therefore, undertaking a comprehensive inquiry into the impact of AI-assisted language learning tools on the language learning achievements of EFL learners would constitute a significant and valuable contribution to the current body of literature. Furthermore, exploring the role of AI-assisted instruction in enhancing EFL learners’ second language (L2) motivation and self-regulated learning, both of which are crucial aspects of English language learning, would provide further depth to the literature. L2 motivation significantly influences English language learners’ engagement and efforts to achieve language proficiency (Boo et al., 2015), while self-regulatory learning pertains to learners’ ability to autonomously plan, monitor, and evaluate their language learning progress (Moyer, 2014).

The present study contributes to the existing literature in several ways. First and foremost, it addresses a notable gap by providing empirical evidence on the specific impact of AI-assisted language learning tools on EFL learners’ language learning achievement, L2 motivation, and self-regulated learning. Although prior research has explored the effectiveness of AI-driven language learning tools, our study narrows its scope to EFL learners, providing valuable insights tailored to this specific context. Second, this research extends the theoretical understanding of the role of AI in language instruction. While the literature has recognized the favorable impacts of AI on language learning, we have adopted a mixed methods research design to delve more profoundly into the specific ways in which AI-mediated instruction contributes to the improvement of language proficiency and the emotional and motivational aspects of EFL learners.

The theoretical contribution of this study extends to the broader understanding of the interplay between technology-driven instruction and pedagogical strategies. The investigation aimed to elucidate whether the observed differences between AI and traditional instruction conditions are attributed to the technology used or the instructional strategies employed. By scrutinizing these elements, the study seeks to provide insights into the distinctive advantages that AI-supported instruction may bring to the language learning landscape, shedding light on whether its impact is rooted in technological novelty or pedagogical innovation.

## 2. Literature review

### 2.1. Theoretical framework

The theoretical framework underpinning this study draws on Vygotsky’s (1984) influential contributions to social constructivist theories of learning. Vygotsky’s work provided a crucial foundation for the development of social constructivism, which emphasizes the significant role of social interactions and collaborative learning experiences and cognitive growth. In this perspective, less proficient learners engage in collaborative learning activities with more proficient individuals, including instructors or, in contemporary settings, computer programs. These interactions serve as cognitive scaffolding, supporting less proficient learners in the acquisition and development of their knowledge (Vygotsky and Cole, 1978; Vygotsky, 1984).

A fundamental concept within social constructivist theory is the Zone of Proximal Development (ZPD), which is proposed in a broader developmental perspective, encompassing various domains beyond learning activities (Vygotsky et al., 1997). The ZPD comprises two distinct levels: the actual level, reflecting a learner’s demonstrated abilities in independent tasks, and the potential level, representing their untapped capacity that can be realized through active engagement in interactive learning activities with peers or more proficient individuals. The ZPD illuminates the dynamic interplay between a learner’s current state of development and the scaffolding provided by the learning environment. It serves as a compass guiding educator to facilitate learning experiences that are optimally challenging, promoting growth and skill acquisition.

In the context of this study, both the AI-assisted and non-AI-assisted groups engage in interactive language learning activities, firmly aligned with Vygotsky’s social constructivist approach (Swain et al., 2015). In the control group, learners interact with their peers, contributing to each other’s ZPD by collaboratively assisting in their language learning journey. Conversely, in the experimental group, learners engage with an AI-assisted language learning tool, which serves as a collaborative partner in the language learning process. Through these interactions, learners harness AI technology to regulate their language learning and advance toward their ZPD, underpinning the core of our research exploration.

In alignment with Vygotsky’s principles, this study incorporates contemporary theories related to AI and computer-assisted learning, recognizing the transformative impact of technology on language instruction. The integration of AI technology introduces additional layers of collaborative learning (Zheng et al., 2021; Weng and Chiu, 2023), where learners interact with AI-assisted language learning tools to regulate their language learning experiences and advance toward their ZPD. These interactions, which parallel the core principles of Vygotsky’s social constructivist theory, emphasize the role of technology as a collaborative partner in the language learning process.

Also, it is worth noting that collaborative abilities emerge as a pivotal aspect that significantly influences the effectiveness of AI-based educational tools in the context of AI-mediated instruction and learning (Akata et al., 2020; Chen et al., 2022). Collaborative abilities refer to learners’ proficiency in actively engaging with AI systems, instructors, or peers to collectively enhance their learning skills and achieve optimal learning outcomes (Hwang et al., 2020). These abilities encompass a spectrum of skills and strategies employed by learners to interact effectively within AI-mediated environments.

Collaborative abilities also play a fundamental role in shaping the dynamics of AI-assisted language learning (Fitria, 2023). Learners who exhibit strong collaborative abilities are adept at harnessing AI tools to engage in meaningful interactions, seek clarification, and co-construct knowledge. Moreover, collaborative abilities are closely intertwined with the utilization of feedback mechanisms within AI systems (Hsu et al., 2023). The quality and impact of feedback received by learners depend on their collaborative abilities to effectively interpret and apply feedback to their language learning practices.

Overall, via integrating Vygotsky’s foundational principles with contemporary theories in AI and computer-assisted learning, this study seeks to explore the evolving dynamics of language learning in the digital age. The collaborative interactions between learners and AI technology might represent a convergence of established educational theories and emerging technological paradigms, contributing to a deeper understanding of language learning achievement, L2 motivation, and self-regulated learning in the context of AI-mediated language instruction.

### 2.2. Artificial intelligence

The rapid advancement of AI has revolutionized various domains, including education, with profound implications for teaching and learning practices (Chen et al., 2020). AI, as a branch of computer science, enables machines to simulate human intelligence, learn from experiences, and perform tasks that typically require human cognitive abilities. In the education contexts, AI technologies hold immense potential to transform traditional instructional methods, providing personalized learning experiences tailored to individual needs and preferences (Hwang et al., 2020). From intelligent tutoring systems and language learning applications to adaptive learning platforms, AI’s integration in education has garnered considerable attention from researchers, educators, and policymakers worldwide (Ilkka, 2018; Kim et al., 2019; Chen et al., 2022; Huang and Tan, 2023).

According to Aldosari (2020), AI is characterized as an intelligent program capable of executing diverse tasks. For instance, individuals can seek assistance from AI-powered tools for academic inquiries, and these tools promptly provide the required information. AI finds applications in educational settings, making intelligent decisions akin to human decision-making. Additionally, AI is widely employed in language learning to enhance learners’ language skills and sub-skills (Zhang and Zou, 2020; Xia et al., 2022a). Numerous AI-assisted language learning tools are accessible on computers and mobile devices, facilitating language learners in their language learning endeavors. These tools offer valuable support in improving various language learning skills.

For instance, ChatGPT, which is an AI-assisted tool, might be utilized in language learning settings to help learners develop their language learning skills and sub-skills (Fang et al., 2023; Fitria, 2023; Kim, 2023; Schmidt-Fajlik, 2023; Su et al., 2023; Yan, 2023). ChatGPT can provide language learners with the required feedback and comments on different language learning skills and sub-skills issues in order to contribute to learners’ language achievement in general. ChatGPT is able to provide grammatically sound sentences which can help learners produce well-organized texts. This AI-assisted language learning tool can also understand human inquiries and provide the best possible answers (Huang and Tan, 2023).

A substantial body of studies has been carried out examining the effects of AI-assisted language learning tools on English language learners’ language achievement (Suryana et al., 2020; Divekar et al., 2022; Fitria, 2023). For instance, Zheng et al. (2021) carried out a meta-analysis of the impact of AI on learning achievement and learning perception. Twenty-four papers, including 2,908 participants, from 2001 to 2020 were analyzed. The results indicated more significant effects of AI on learning achievement in comparison to its effects on learning perception. This means that most of the studies revolved around the great impact of AI on learning achievement followed by its impact on learner perceptions. Xu et al. (2022) examined the effects of AI-assisted language learning on English language learner speech and interaction. The findings indicated that the AI-assisted language learning tool using a speech recognition feature improved the learners’ language learning achievement and engaged them in interactive language learning activities.

Ebadi and Amini (2022) investigated the impact of AI-assisted language learning on EFL learners’ language learning engagement. The data were collected via motivation, social presence, and human-likeness questionnaires and recording the interaction of the EFL learners with the AI tool. The findings revealed that the AI tool had a significant impact on the learners’ learning motivation and engagement. In a similar vein, Carpio Cañada et al. (2015) examined the effects of an AI-powered language learning approach on language learners’ motivation and learning achievement. The results indicated that the AI-assisted language learning approach contributed to the learners’ language learning motivation. The learners’ motivation in language learning activities also resulted in the learners’ better learning achievement. Similarly, Ali et al. (2023) explored the impact of an AI tool (i.e., ChatGPT) on English language learners’ and teachers’ motivation. The results demonstrated that the AI-assisted language learning tool had a great contribution to the learners’ writing and reading skills, while it had neutral influences on their speaking and listening skills. In much the same vein, Schmidt-Fajlik (2023) compared the differences between ChatGPT, Grammarly, and ProWritingAid tools in checking, understanding, and developing EFL learners’ English grammar. The findings indicated that ChatGPT was more effective than the other AI tools in detecting and improving the EFL learners’ English grammar.

As for language learning sub-skills, Hsu et al. (2023), for example, examined the impact of AI-assisted image recognition technologies on EFL learners’ vocabulary knowledge, self-regulation, and anxiety. Utilizing an experimental research design, the learners in the experimental group received AI-assisted image recognition technologies to demonstrate images along with the related vocabulary to develop the learners’ vocabulary knowledge. The findings showed that the experimental learners developed their vocabulary knowledge and self-regulation and decreased their language learning anxiety. However, the experimental learners only outperformed their control counterparts in vocabulary knowledge. That is, there were no significant differences between the experimental and control groups regarding their self-regulation and language learning anxiety. Kim (2019) also investigated the influence of AI-supported language learning instruction on EFL university students’ grammar skills. Using an experimental research design, one group of the students received the AI tool while the other group followed the conventional instruction of grammar skills. The results showed that the experimental students who received the AI-based instruction outperformed those who did not receive the AI tool, signifying that the AI-assisted language learning instruction had a great contribution to the students’ grammar skill development.

With regard to language learning skills, Yan (2023), for instance, examined the role of an AI-assisted language learning tool on EFL students’ writing performance. The findings revealed that the AI-assisted language learning tool developed the writing performance of the EFL learners. Yan also claimed that the EFL learners, using the AI-assisted language learning tool, could accomplish their writing tasks more swiftly than ever, findings which capitalized on the efficient role of the AI-assisted language learning tool for the writing tasks. Similarly, Utami et al. (2023) investigated the impact of AI-powered language learning on the academic research writing of three Indonesian EFL learners. Following a case study research design, the required data were gathered via questionnaires and interviews. The results showed that the AI-powered language learning approach enhanced the students’ academic research writing by giving them the required feedback, comments, and alternative sentences. Moreover, the AI-powered language learning approach was found to enhance the writing engagement of the EFL learners. Lee et al. (2023) also explored the effects of an AI-assisted language learning tool on EFL learners’ reading enjoyment. A group of EFL learners received the AI tool to generate reading topics for the learners based on their interests, while the other group were engaged in conventional reading comprehension activities. The findings revealed that the AI-powered language learning tool had a substantial contribution to the learners’ reading enjoyment.

El Shazly (2021) investigated the impact of AI-powered language learning tools on Egyptian EFL learners’ speaking performance and foreign language anxiety. The learners’ speaking performance was examined through roleplaying activities and evaluated via an IELTS-speaking test, and their foreign language anxiety was examined through a questionnaire. The findings revealed that the use of the AI-assisted language applications in the learners’ speaking activities did not diminish the learners’ foreign language anxiety. However, it was revealed that the utilization of AI-assisted language learning tools increased the learners’ speaking performance and interactive speaking activities. In much the same vein, Junaidi (2020) investigated the impact of AI-assisted language learning tools on EFL learners’ speaking skills. Adopting a quasi-experimental research design, both experimental and control groups’ speaking skills, including fluency, grammar, vocabulary, and pronunciation, were assessed via pre- and post-tests to check the learners’ improvements in their speaking skills on the one hand and to examine the differences between the experimental and control learners’ speaking skills on the other hand. The findings demonstrated that the learners’ speaking skills were developed in both groups; however, the experimental learners outperformed their control counterparts in speaking skills, which corroborated the significant roles of AI-powered language learning tools in developing EFL learners’ speaking skills.

The literature review presented herein illuminates the multifaceted impact of AI-assisted language learning tools on various language skills and sub-skills, underscoring their potential to enhance vocabulary knowledge, grammar proficiency, writing skills, reading enjoyment, speaking performance, as well as learners’ motivation and engagement. Although previous research has probed the effects of AI on specific facets of language learning, a noteworthy gap exists in the exploration of its holistic influence on overall language achievement, particularly in the context of EFL instruction for Chinese learners. Moreover, the dimensions of L2 motivation and self-regulated learning, integral components of language proficiency and autonomous learning, have received limited attention within the realm of AI-supported language instruction. This discernible gap in the literature paves the way for the current study, which not only aims to bridge this void but also to elucidate whether the observed differences between AI and traditional instruction conditions stem from technological novelty or pedagogical innovation. Thus, the present research seeks to contribute to the broader understanding of technology-driven language instruction and its multifaceted implications, shedding light on AI’s potential to reshape language learning paradigms.

### 2.3. The present study

As supported by the existing literature, AI-assisted language learning applications have demonstrated a significant role in enhancing English language learners’ overall language learning achievement and specific language skills and sub-skills (Kim, 2019; El Shazly, 2021; Xu et al., 2022; Lee et al., 2023; Schmidt-Fajlik, 2023; Yan, 2023). Moreover, these studies have shown that AI-powered language learning tools contribute to improved self-regulated learning and increased motivation among English language learners (Carpio Cañada et al., 2015; Ebadi and Amini, 2022; Hsu et al., 2023).

Despite the positive findings from the literature, there remains a dearth of research specifically investigating the impact of AI-assisted language learning tools on EFL learners’ English learning achievement, L2 motivation, and self-regulated learning. To address this gap, the present study aimed to quantitatively examine the effects of the AI-assisted language learning approach on these key aspects of EFL learners’ language development. Additionally, in order to gain deeper insights into the learners’ experiences, perceptions, and attitudes toward the utilization of AI-powered language learning tools, the study also conducted a qualitative exploration.

The research questions guiding this study are as follows:

1. Are there any significant differences between AI and non-AI-assisted language learning instruction in developing English learning achievement, L2 motivation, and self-regulated learning of EFL learners?
2. What are the perceptions of EFL learners toward the effects of AI-assisted language learning on their language learning achievement?

## 3. Method

### 3.1. Participants

This study was conducted at a university in mainland China, involving a carefully selected group of EFL learners. Two intact classes (*n* = 60) were included, and participants were randomly assigned to either the experimental or control group to ensure a fair distribution of learners’ characteristics. The Experimental Group consisted of 30 participants, comprising 17 females and 13 males, all aged between 19 and 23 years, with intermediate English proficiency levels, as assessed through a standardized English proficiency test. Similarly, the Control Group consisted of 30 participants, including 19 females and 11 males, aged between 20 and 24 years, with intermediate English proficiency levels evaluated through the same standardized test.

To be included in the study, participants had to meet certain criteria, including being undergraduate students majoring in various fields at the university, having no prior experience with AI-mediated language instruction, and not being diagnosed with any learning disabilities that could significantly affect their language learning abilities.

Throughout the study, ethical considerations were of utmost importance to ensure the well-being and rights of the participants. Before the study began, informed consent was obtained from all participants, ensuring they were fully informed about the purpose and procedures of the research, and they had the right to withdraw at any time. The researchers also maintained strict confidentiality and data anonymity to protect the participants’ privacy. The study adhered strictly to ethical guidelines for research involving human participants, highlighting the researchers’ commitment to prioritize the participants’ well-being and rights at every stage of the study.

### 3.2. Instruments

#### 3.2.1. English achievement test

To gauge participants’ initial level of English achievement, a comprehensive English achievement test was administered to both the experimental and control groups during the pre-test phase. The English achievement test was meticulously designed by a panel of experienced teachers with extensive expertise in language teaching and testing, ensuring its reliability and relevance to the study’s objectives. The test was thoughtfully tailored to align with the learning objectives and curriculum of the university’s EFL program, making it a suitable tool to assess participants’ language proficiency levels accurately. It consisted of multiple sections, each evaluating essential language skills, including grammar, vocabulary, reading comprehension, and writing. To ensure the quality and validity of the English achievement test, the assessment underwent a thorough evaluation by three domain experts. Their valuable input and critical analysis confirmed its face and content validity, ensuring that the test effectively measured the targeted language competencies. The English achievement test demonstrated high reliability with a Cronbach alpha coefficient of 0.87, indicating strong internal consistency among its items.

#### 3.2.2. L2 motivation scale

In this study, students were given the 16-item motivation scale designed by Mehdiyev et al. (2017) at both the beginning and end of the procedure process. The scale utilizes a five-point Likert scale, ranging from ‘Totally agree’ to ‘Absolutely disagree’. It comprises three distinct factors: attitude (4 items, “I find learning English more challenging compared to others.”), self-confidence (5 items, “The process of learning English gives me happiness.”), and personal use (7 items, “I aim to acquire English skills to stay informed about global developments.”). The Cronbach alpha values for each factor were found to be 0.81, 0.79, and 0.82, respectively, with an overall reliability coefficient of 0.82.

#### 3.2.3. Self-regulation questionnaire (SRQ)

The Self-Regulation Questionnaire (SRQ) utilized in this study was developed by Brown et al. (1999). It consists of 63 items designed to assess seven subprocesses, forming seven rationally derived subscales of the SRQ. Each subscale comprises nine items, and the total sum score is recommended to estimate the participants’ self-regulation ability. To respond to the items, students in the study used a Likert-type scale ranging from 1 (strongly disagree) to 5 (strongly agree). A sample item of the scale is “I can effectively manage my study time to meet my learning goals.” In the pilot study conducted for this research, the SRQ exhibited a reliability index of 0.83, further confirming its consistency and accuracy.

#### 3.2.4. Semi-structured interview

To collect qualitative data, we conducted semi-structured interviews with 14 participants from the experimental group. We employed a purposive sampling strategy to ensure a well-rounded representation of both gender and age groups within the experimental cohort, aiming to capture a diverse range of experiences and perspectives for a richer qualitative analysis. The demographic profile of these participants is as follows: Six of them were female, while eight were male, with ages ranging from 20 to 24 years. Their English proficiency levels were assessed as intermediate through standardized tests conducted at the study’s outset. Furthermore, the participants brought diverse academic backgrounds, spanning various disciplines across the university’s academic spectrum.

The semi-structured interviews were thoughtfully designed to offer participants a flexible yet focused platform to share their insights and perceptions regarding AI-mediated language instruction. These interviews explored a wide array of aspects related to their language learning journey with the Duolingo platform. Topics encompassed their emotions and attitudes toward interactive learning activities, the influence of personalized feedback on language skill development, and their levels of motivation and autonomy throughout the learning process. Additionally, participants were encouraged to reflect on any challenges they encountered and articulate the advantages they perceived from their engagement with AI-mediated instruction. Conducted in a one-on-one format, these interviews fostered a comfortable and open atmosphere that facilitated participants in freely expressing their thoughts. With their consent, each interview session was audio-recorded, supplemented by detailed notes capturing both verbal and non-verbal cues, as well as contextual information. Subsequently, we transcribed the interview transcripts verbatim and subjected them to a rigorous thematic analysis, a systematic method used to identify and analyze meaningful patterns and themes within the qualitative data.

#### 3.2.5. AI-mediated language learning platform

For the experimental group, the AI-mediated language learning platform, Duolingo, was utilized to deliver interactive and personalized English language lessons. Duolingo’s effectiveness in providing adaptive and engaging language instruction has been widely recognized, making it a suitable platform for this study. Learners in the experimental group had access to a variety of interactive exercises, online quizzes, language games, and real-time feedback tailored to their individual performance on the platform. The control group received traditional language instruction using standard teaching materials, including textbooks, lectures, and classroom activities. Teacher-led discussions, grammar drills, reading passages, and writing exercises were incorporated into the curriculum to facilitate language learning in the control group.

#### 3.2.6. Data collection procedure

Before implementing the intervention, a pre-test assessment was conducted for both the experimental and control groups to measure their initial English learning achievement. The pre-test evaluated participants’ proficiency in grammar, vocabulary, reading comprehension, and writing skills. Additionally, participants completed self-report questionnaires to gauge their L2 motivation and self-regulated learning strategies, providing valuable insights into their attitudes and approaches toward language learning. Once the pre-test phase was completed, random assignment was used to divide participants into either the experimental group or the control group. This random assignment ensured that any individual differences or biases between the two groups were evenly distributed, enhancing the study’s reliability.

The experimental group was exposed to AI-mediated language instruction using the renowned AI-powered language learning platform, Duolingo. The 10-week intervention period included interactive and personalized English language lessons. Participants engaged in various activities, such as interactive exercises, online quizzes, language games, and received real-time feedback tailored to their performance. In-class activities involved group discussions, language exercises, and practice sessions with Duolingo, all facilitated by the instructor. To reinforce learning, participants were encouraged to use Duolingo outside of class, dedicating at least 2 h per week to supplementary practice.

In parallel, the control group received traditional language instruction for the same 10-week duration. This instruction emphasized conventional teaching methods and materials, including textbooks, lectures, and classroom activities. Sample activities included teacher-led discussions, grammar drills, reading passages, and writing exercises. In-class sessions were teacher-centered, with the instructor leading the lectures and activities. Outside of class, participants were encouraged to practice through assigned homework, dedicating a minimum of 2 h per week to further develop their language skills.

After the 10-week intervention period, both groups underwent a post-test assessment mirroring the pre-test evaluation. The post-test measured English learning achievement, L2 motivation, and self-regulated learning, enabling a comparative analysis of the two groups’ progress and the impact of their respective interventions.

Throughout the data collection process, researchers diligently monitored the implementation of interventions and participant engagement to ensure accuracy and validity. Ethical considerations, including obtaining informed consent and maintaining confidentiality, were strictly adhered to throughout the study to protect the participants’ rights and well-being. The data collected from the pre-test and post-test assessments, as well as the self-report questionnaires, were subsequently subjected to appropriate statistical analyses to determine the effects of AI-mediated language instruction using Duolingo on English learning achievement, L2 motivation, and self-regulated learning when compared to traditional language instruction.

### 3.3. Data analysis

#### 3.3.1. Quantitative data analysis

For the quantitative phase, descriptive statistics and inferential analyses examined the effect of AI-mediated language instruction on English learning achievement, L2 motivation, and self-regulated learning of EFL learners. Statistical Package for the Social Sciences (SPSS) version 26 was used for all analyses. Descriptive statistics summarized pre-test and post-test scores for both groups. A mixed-design analysis of variance (ANOVA) was conducted to determine the intervention effect, assessing main effects of group (experimental vs. control), time (pre-test vs. post-test), and the interaction between group and time on L2 achievement, L2 motivation, and self-regulation. The main effect of group compared post-test scores, indicating AI-mediated instruction’s impact on these variables compared to traditional instruction. The interaction effect between group and time explored differences in impact over the intervention period.

#### 3.3.2. Qualitative data analysis

For the qualitative phase, thematic analysis examined semi-structured interview data from 14 students in the experimental group. Transcripts were transcribed verbatim and subjected to open coding, identifying initial codes capturing participants’ experiences with AI-mediated instruction. Codes were organized into potential themes, reflecting meaningful patterns related to the impact on English learning achievement, L2 motivation, and self-regulated learning. Thematic saturation was achieved through iterative coding, and member checking and peer debriefing ensured trustworthiness (Williams and Morrow, 2009). Integrating qualitative findings with quantitative results in the discussion section provided a comprehensive understanding of AI-mediated language instruction’s impact. Triangulation enriched the analysis, enhancing validity and reliability.

## 4. Results

### 4.1. Impact on English learning achievement, L2 motivation, and self-regulated learning

Prior to conducting the mixed-design ANOVA, we assessed the assumptions for robust and reliable inferential statistics. Normality assumption was examined through histograms, Q-Q plots, and Shapiro–Wilk tests, which confirmed that the data were approximately normally distributed within each group and time point (*p* > 0.05). The assumption of homogeneity of variance was met as indicated by Levene’s test and Brown-Forsythe test (*p* > 0.05) for all conditions. Sphericity assumption was violated for the repeated measures factor (Time) in the analysis of L2 achievement (*p* < 0.05); thus, the Greenhouse–Geisser correction was applied. The assumption of independence was satisfied through random assignment of participants to experimental and control groups, ensuring data collection without external influence.

Table 1 presents the descriptive statistics of the pre- and post-test scores of L2 achievement, L2 motivation, and self-regulation for both the experimental and control groups. Prior to the intervention, there were no significant differences between the two groups in terms of their pre-test scores for L2 achievement (Experimental: *M* = 43.21, *SD* = 12.69; Control: *M* = 44.39, *SD* = 13.21), L2 motivation (Experimental: *M* = 3.12, *SD* = 1.6; Control: *M* = 3.04, *SD* = 1.4), and self-regulation (Experimental: *M* = 2.89, *SD* = 0.54; Control: *M* = 3.01, *SD* = 0.69). These similar baseline scores ensured a balanced starting point for the intervention.

**Table 1:** Descriptive statistics of pre- and post-test scores of L2 achievement, L2 motivation, and self-regulation in both experimental and control groups.

		Pre-test		Post-test	
	Group	M	SD	M	SD
L2 Achievement	Experimental	43.21	12.69	73.86	15.26
	Control	44.39	13.21	61.11	14.97
L2 Motivation	Experimental	3.12	1.6	3.89	1.8
	Control	3.04	1.4	3.35	1.5
Self-regulation	Experimental	2.89	0.54	3.94	0.73
	Control	3.01	0.69	3.37	0.76

Following the 10-week intervention period, the experimental group (*M* = 73.86, *SD* = 15.26) demonstrated a significant improvement in their post-test scores on L2 achievement compared to the control group (*M* = 61.11, *SD* = 14.97). The mixed-design ANOVA results in Table 2 revealed a significant main effect for Group [*F*(1, 58) = 94.35, *p* < 0.001, *η*^2^ = 0.81], indicating that the AI-mediated language instruction had a substantial impact on enhancing English learning achievement among EFL learners. Moreover, there was a significant main effect for Time [*F*(1, 58) = 10.89, *p* < 0.001, *η*^2^ = 0.27], suggesting that both groups experienced an overall improvement in their L2 achievement scores over the 10-week period. Additionally, the interaction effect between Group and Time was significant [*F*(1, 58) = 8.37, *p* < 0.001, *η*^2^ = 0.23], indicating that the improvement in L2 achievement significantly differed between the experimental and control groups as a result of AI-mediated instruction.

**Table 2:** Mixed-design ANOVAs, investigating the differences between the two groups’ post-test scores on L2 achievement, L2 motivation, and self-regulation.

Source		Type III sum of squares	df	Mean square	F	Sig.	Partial eta squared
L2 achievement	Group	836.18	1	836.18	94.35	0.00	0.81
	Time	73.12	1	73.12	10.89	0.00	0.27
	Group × Time	56.54	1	56.54	8.37	0.00	0.23
L2 Motivation	Group	16.56	1	16.56	9.76	0.00	0.18
	Time	53.12	1	53.12	34.10	0.00	0.44
	Group × Time	10.91	1	10.91	7.43	0.04	0.11
Self-regulation	Group	82.54	1	82.54	11.36	0.00	0.64
	Time	21.12	1	21.12	8.94	0.00	0.24
	Group × Time	13.85	1	13.85	7.68	0.00	0.38

Regarding L2 motivation, the experimental group (*M* = 3.89, *SD* = 1.8) exhibited a significant increase in their post-test scores compared to the control group (*M* = 3.35, *SD* = 1.5) after the intervention. The mixed-design ANOVA results in Table 2 indicated a significant main effect for Time [*F*(1, 58) = 34.10, *p* < 0.001, *η*^2^ = 0.44], suggesting that both groups experienced an overall improvement in their motivation to learn English over the 10-week period. Nonetheless, there was a significant interaction effect between Group and Time [*F*(1, 58) = 7.43, *p* = 0.04, *η*^2^ = 0.11], indicating that that the improvement in L2 motivation significantly differed between the experimental and control groups due to the AI-mediated instruction.

For self-regulated learning, the experimental group (*M* = 3.94, *SD* = 0.73) demonstrated a significant increase in their post-test scores, whereas the control group (*M* = 3.37, *SD* = 0.76) exhibited a smaller improvement. The mixed-design ANOVA results in Table 2 showed a significant main effect for Group [*F*(1, 58) = 11.36, *p* < 0.001, *η*^2^ = 0.64], indicating that the AI-mediated language instruction had a substantial impact on enhancing self-regulated learning among EFL learners. Moreover, there was a significant main effect for Time [*F*(1, 48) = 8.94, *p* < 0.001, *η*^2^ = 0.24], suggesting that both groups experienced an overall improvement in their self-regulation strategies over the 10-week period. Furthermore, the interaction effect between Group and Time was significant [*F*(1, 58) = 7.68, *p* < 0.001, *η*^2^ = 0.38], indicating that the enhancement in self-regulated learning substantially differed between the experimental and control groups as a result of AI-mediated instruction.

Overall, the findings of this study demonstrate that the AI-mediated language instruction significantly enhanced English learning achievement, L2 motivation, and self-regulated learning among EFL learners. Although both groups experienced an increase in L2 achievement, motivation, and self-regulation over time, the AI-mediated instruction showed a significant advantage over traditional instruction in terms of the dependent variables. These results highlight the potential of AI-based language learning platforms, such as the one used in the experimental group, to positively impact language learning outcomes, motivation, and foster self-regulated learning strategies among EFL learners.

### 4.2. Learners’ perceptions

To gain a comprehensive understanding of the experiences and perceptions of students who participated in the AI-mediated language instruction, a qualitative phase was integrated into the study. Semi-structured interviews were conducted with 14 carefully selected students from the experimental group, ensuring a diverse range of perspectives and experiences. These interviews aimed to delve deeper into the nuances and subjective aspects of the students’ engagement with the AI-mediated instruction, shedding light on the underlying mechanisms and contextual factors that influenced their English learning achievement, L2 motivation, and self-regulated learning.

#### 4.2.1. Thematic analysis: emergent themes

Thematic analysis was employed to identify and analyze the emergent themes that encapsulated the rich insights shared by the interview participants. The themes that emerged from the interviews revealed profound insights into the impact of AI-mediated language instruction on the students’ language learning journey. The following themes were identified.

#### 4.2.2. Engaging and immersive learning experience

Participants were notably enthusiastic about the impact of AI-mediated language instruction, often describing it as a refreshing and captivating learning experience. They conveyed how the interactive nature of the AI platform’s exercises, combined with dynamic language games and immediate feedback, created an immersive atmosphere that held their attention and made learning enjoyable. One participant shared, “Using the AI platform felt like I was actively participating in my learning. The interactive exercises were like puzzles I wanted to solve, and the real-time feedback kept me engaged.”

The platform’s ability to transform language learning into an interactive adventure was frequently highlighted. Students expressed that the AI platform’s engaging features turned the learning process into a captivating journey, making it an experience they looked forward to. One participant noted, “Learning with the AI platform was exciting. It wasn’t just about reading textbooks; it was like diving into a world of English where I could explore and interact.”

#### 4.2.3. Tailored and personalized learning pathway

Students recognized and valued the AI platform’s capability to tailor the learning content to their individual strengths, weaknesses, and proficiency levels. They emphasized that the platform’s personalized approach offered them a customized learning pathway that focused on their specific needs. This tailored instruction enabled them to address their challenges more effectively and capitalize on their strengths. One participant elaborated, “The AI platform did not treat everyone the same. It understood where I needed improvement and guided me accordingly.”

This personalized learning experience contributed to participants’ sense of autonomy and confidence in their language learning journey. They expressed how the platform’s recognition of their learning style and preferences created an environment where they felt in control of their progress. As one other student pointed out, “The AI platform adapted to my pace and style. It pushed me when I was ready for a challenge and provided support when I needed it. It felt like I had a personalized tutor.”

#### 4.2.4. Significant improvement in language proficiency

Participants attributed their significant advancements in English language proficiency to the AI-mediated instruction. They highlighted how the interactive and adaptive features of the AI platform contributed to their enhanced skills across multiple language dimensions, including grammar, vocabulary, reading comprehension, and writing. Several students shared personal anecdotes of their progress, illustrating how their newfound language abilities positively impacted various aspects of their academic and personal lives.

The students’ stories underscored how their improved language proficiency enabled them to excel in coursework that required English language skills, leading to higher grades and increased academic confidence. One participant recounted, “I used to struggle with writing essays, but after using the AI platform, my writing skills improved. This semester, I received one of my highest essay scores.”

#### 4.2.5. Intrinsic motivation and active engagement

The AI-mediated instruction played a transformative role in cultivating intrinsic motivation among students and driving active engagement with the English language. Participants described a heightened sense of curiosity and enthusiasm that extended beyond the classroom setting. Many of them took proactive measures to seek out supplementary language resources and opportunities for practice. The AI platform, by fostering a genuine interest in English, inspired a passion for continuous exploration and learning.

The impact of this intrinsic motivation was evident in the participants’ dedication to continuous language practice and their willingness to invest extra effort in their language learning journey. One participant expressed, “The AI platform ignited a spark in me. I wasn’t just learning because I had to; I genuinely wanted to improve and discover more about the language.”

#### 4.2.6. Empowerment in self-regulated learning

Participants reported a notable sense of empowerment in their self-regulated learning practices, largely attributed to the guidance provided by the AI platform. They shared how the platform facilitated their use of various self-regulation strategies, such as setting clear learning goals, planning study sessions, and regularly monitoring their progress. The AI platform acted as a dependable companion, offering structure and tools that empowered participants to navigate their learning paths with confidence.

The participants’ testimonials highlighted how the AI platform’s guidance transformed them into more autonomous learners. One student reflected, “With the AI platform, I learned how to set specific goals and manage my learning time effectively. I felt more in control of my progress.” Another student noted, “The AI platform helped me become a more independent learner. It taught me how to set goals and take ownership of my learning process.”

#### 4.2.7. Positive learning environment and alleviated anxiety

Participants felt that the AI-mediated instruction played a crucial role in cultivating an environment conducive to positive learning experiences. They emphasized how the platform’s design encouraged them to step out of their comfort zones, take risks, and embrace mistakes without fear of judgment. This inclusive atmosphere was described as a catalyst for enhanced learning. One participant shared, “I used to hesitate to speak English, fearing I’d say something wrong. But with the AI platform’s support, I felt safe to express myself. It really helped boost my confidence.”

Moreover, the non-judgmental and constructive feedback provided by the AI platform was highlighted as a key factor in mitigating anxiety. Participants revealed that the platform’s feedback not only pointed out their errors but also offered explanations and suggestions for improvement. This approach allowed them to learn from their mistakes and develop resilience in their language learning process. As one participant noted, “When I got something wrong, the AI would explain why and show me how to get it right. It made me feel like mistakes were just part of the learning process.”

#### 4.2.8. Flexibility and convenience for language practice

Participants enthusiastically highlighted the flexibility and convenience offered by the AI platform for practicing English. They appreciated the freedom to engage in language learning at their own pace, regardless of time or location. Many mentioned that they could seamlessly integrate language practice into their daily routines, such as during commutes or breaks. One participant shared, “I could practice English whenever I had a spare moment. It made learning feel like a part of my life, not an extra task.”

The AI platform’s accessibility beyond the traditional classroom setting was noted as a significant advantage. Participants emphasized that this flexibility allowed them to maintain consistent language practice even outside of formal study hours. They praised the platform for enabling them to stay engaged with English without the constraints of physical space. A participant explained, “With the AI platform, I did not have to wait for a class or find a specific place to study. It gave me the freedom to learn wherever I was.”

The integration of the qualitative phase, with its in-depth interviews and thematic analysis, complemented the quantitative findings by providing rich, nuanced insights into the students’ experiences with AI-mediated language instruction. The emergent themes highlighted the positive impact of the AI platform on their learning journey, emphasizing the importance of engaging and personalized instruction, intrinsic motivation, self-regulated learning, positive learning environments, and flexible language practice. These qualitative findings enhance the overall understanding of the effectiveness and benefits of AI-mediated language instruction for EFL learners, contributing to a more comprehensive and nuanced understanding of the phenomenon.

## 5. Discussion

This research investigated the efficacy of AI-assisted language learning instruction in enhancing English learning achievement, L2 motivation, and self-regulated learning among Chinese EFL learners. Drawing on Vygotsky’s (1984) social constructivist theory of learning, a mixed-methods approach was employed for comprehensive data collection and analysis. The quantitative findings demonstrated a significant positive impact of AI-powered language learning instruction on the EFL learners’ English learning achievement, L2 motivation, and self-regulated learning. Specifically, the AI-powered instruction was found to effectively improve all aspects of language learning achievement, including grammar, vocabulary, reading comprehension, and writing skills.

These quantitative results align with previous research conducted by Xu et al. (2022) and Zheng et al. (2021), who also reported positive outcomes of AI-powered language learning instruction on EFL learners’ learning achievement. Additionally, the current study’s findings are consistent with the research conducted by Hsu et al. (2023) and Utami et al. (2023), which highlighted the significant impact of AI-assisted language learning on language learners’ vocabulary knowledge and writing performance, respectively.

In accordance with Vygotsky’s (1984) social constructivist theory of learning, the AI learners were initially immersed in collaborative language learning activities facilitated by the AI-assisted language learning tool, with the primary goal of enhancing their language learning achievement in grammar, vocabulary, reading comprehension, and writing skills. These collaborative activities, which encompassed elements of other-regulation, played a pivotal role in assisting learners in regulating their cognitive abilities related to language achievement, leading to the gradual internalization of their learning capabilities (Swain et al., 2015; Kim et al., 2018). In other words, the AI tool served as a catalyst, engaging learners in collaborative language learning activities that ultimately fostered the internalization of these skills (Zheng et al., 2021; Xu et al., 2022). While learners in the non-AI class also internalized their language learning abilities through interactions with peers in collaborative activities, the AI learners demonstrated superior performance due to their utilization of the AI-assisted language learning tool. The AI system expedited the transition from other-regulation to self-regulation of language learning abilities, enabling AI learners to reach their ZPD or potential level of functioning earlier than anticipated (Davis and Miyake, 2018). These findings align with the qualitative insights from the study, as learners corroborated the positive impact of the AI-assisted language learning tool on their independent functioning in language learning activities (Liang et al., 2021). The collaborative aspect of these activities, intertwined with the AI tool’s support, played a crucial role in this transition.

Moreover, the student-centered nature of language learning activities adopted in both classes likely contributed to the observed outcomes. Both groups engaged in group-work activities, while the teacher assumed the role of facilitator, encouraging students to partake in collaborative language learning endeavors. These student-centered activities positively influenced the language achievement of EFL learners in both groups (Levine, 2004). However, the integration of the AI-assisted language learning tool in the AI learners’ collaborative activities appeared to be more effective in enhancing their learning achievement, synergizing the benefits of collaborative activities with the adaptive supports provided by the AI tool. This can be attributed to the AI tool’s ability to provide personalized feedback and comments on learners’ language skills, which significantly impacted their language achievement (Kim et al., 2019; Liang et al., 2021; Chen et al., 2022). The AI system offered more effective and efficient language learning feedback, comments, and alternatives compared to other language learners, thus making a substantial contribution to learners’ language achievement (Dodigovic, 2007; Kim et al., 2019). Although collaborative activities played a crucial role in language learning, it was the combination of collaborative activities and the adaptive supports provided by the AI tool that seemed to have the most significant impact on learners’ language achievement. The AI learners also benefited from receiving more immediate and specific feedback tailored to their own written texts. These findings further align with the qualitative insights, where learners emphasized that the AI tool provided relevant feedback and facilitated their language skill improvement at their individual pace.

The high level of L2 motivation observed in AI learners may be attributed to their active engagement in interactive language learning activities facilitated by the AI system, enabling them to improve their skills at their own pace (Hwang et al., 2020; Liang et al., 2021). This finding aligns with previous research by Utami et al. (2023), who also reported strong motivation among language learners using AI-powered language learning activities. The qualitative findings of our study further corroborate this aspect, as participants emphasized the motivating language learning activities provided by the AI tool. The user-friendly environment created by the AI tool not only reduced language learning anxiety but also enhanced learners’ L2 motivation (Spiro et al., 2017; Hsu et al., 2023). By offering a personalized language learning environment free from time and space restrictions, the AI tool fostered a sense of autonomy and enthusiasm in learners, thereby contributing to heightened motivation (Carpio Cañada et al., 2015).

In addition, one other significant outcome of this study is the evidence supporting the enhancement of self-regulated learning among Chinese EFL learners through the utilization of AI-assisted language learning instruction. Self-regulated learning, characterized by learners’ ability to independently plan, monitor, and evaluate their learning process, plays a pivotal role in fostering autonomy and lifelong learning skills (Zimmerman, 2000). The integration of AI-mediated instruction has proven to be instrumental in transforming the SRL landscape, offering learners adaptive tools that empower them to take charge of their own learning process (Xia et al., 2022b; Jin et al., 2023; Wang and Lin, 2023).

The qualitative and quantitative findings of this study converge to illuminate the positive impact of AI-assisted instruction on learners’ self-regulatory abilities. Through the personalized nature of the AI platform, learners were presented with tailored learning content, real-time feedback, and opportunities for self-assessment. These features not only catered to learners’ individual strengths and areas requiring improvement but also facilitated the development of their metacognitive and self-regulatory skills (Zimmerman, 1989). The AI tool acted as a dynamic guide, assisting learners in setting realistic goals, strategizing their study sessions, and reflecting on their progress, thereby cultivating their self-regulation capacities.

The thematic analysis of the qualitative data further underscores the enhancement of self-regulated learning attributed to the AI-assisted instruction. Participants vividly expressed how the AI platform empowered them to assume greater control over their learning process. They articulated using goal-setting techniques, tracking their own advancement, and adjusting their learning strategies based on the AI feedback. This increased sense of autonomy and ownership resonates with the core principles of self-regulated learning, as learners transitioned from being passive recipients to active agents in their language learning endeavors (Boekaerts and Corno, 2005; Fathi et al., 2021).

The findings of this study align with the broader educational research highlighting the interplay between technology-enhanced learning environments and self-regulated learning (Steffens, 2006; Johnson and Davies, 2014; Fathi et al., 2019; Lau and Jong, 2022). AI-mediated instruction acts as a scaffold, supporting learners as they navigate through the intricacies of self-regulation. The AI system provides learners with timely insights into their progress and performance, enabling them to make informed decisions regarding their learning strategies and goals (Jin et al., 2023; Wang and Lin, 2023).

The overall findings can be attributed to the efficacy and efficiency of the data generation by the AI tool compared to traditional language teachers’ instruction. AI’s ability to generate immediate and informative feedback proved invaluable to English language learners, addressing their weaknesses and providing timely guidance (Divekar et al., 2022). This prompt feedback mechanism not only supplied learners with essential information but also facilitated the improvement of their language learning skills. Additionally, the availability of written texts and materials for learners’ reference further consolidated their language learning progress, while the flexibility of accessing the materials anytime and anywhere amplified their learning experience (Junaidi, 2020; Hsu et al., 2023).

Also, the qualitative phase of this study, comprising semi-structured interviews with 11 students from the experimental group, provided a deeper understanding of the students’ experiences and perceptions regarding AI-mediated language instruction. Thematic analysis revealed emergent themes that shed light on the multifaceted impact of the AI platform on English learning achievement, L2 motivation, and self-regulated learning among EFL learners. Overall, the findings from the semi-structured interviews align with the quantitative results, reinforcing the positive impact of AI-mediated instruction on language learning. The students’ overwhelming enthusiasm for the engaging and interactive learning experience offered by the AI platform resonates with the observed higher levels of L2 motivation in the experimental group. The platform’s ability to adapt and personalize learning content according to each student’s needs and proficiency level aligns with the significant improvement in language proficiency demonstrated by the experimental group compared to the control group.

The emergent themes collectively emphasize the transformative nature of AI-mediated language instruction (Kim et al., 2019), supporting the existing literature regarding the potential advantages of AI in education in general (e.g., Malik et al., 2019; Chen et al., 2020; Luan et al., 2020; Ouyang and Jiao, 2021). The AI platform acted as more than just a language-learning tool; it became a catalyst for intrinsic motivation, empowerment in self-regulated learning, and the creation of a positive and supportive learning environment. Students reported feeling more at ease taking risks and making mistakes, fostering a growth mindset that further fueled their language learning journey. The interviews also shed light on the convenience and flexibility offered by the AI platform, allowing students to engage in consistent language practice beyond the confines of traditional classroom settings. This continuous and meaningful language practice is reflected in the experimental group’s overall progress and higher English learning achievement compared to the control group.

The integration of qualitative insights enriches the discussion and strengthens the study’s validity by offering a comprehensive view of the students’ experiences with AI-mediated language instruction. The qualitative phase provides nuance and depth, capturing the subjective aspects of the learning journey that quantitative measures may not fully capture. Moreover, the integration of both qualitative and quantitative data enhances the study’s credibility and contributes to a holistic understanding of the phenomenon.

Overall, the study’s findings underscore the significant role of AI-mediated language instruction in enhancing L2 motivation and language learning skills. The interactive and personalized learning environment created by the AI tool empowers learners, reduces anxiety, and fosters intrinsic motivation. Moreover, the prompt feedback mechanism and easy access to learning materials contribute to the overall effectiveness and efficiency of AI-based language learning, making it a valuable asset in modern language classrooms.

The findings have significant implications for both EFL learners and teachers, shedding light on the potential impact of AI-assisted language learning tools in language classrooms.

For EFL learners, the study recommends embracing AI-powered language learning tools as valuable resources to enhance their language learning journey. These tools offer a more engaging and personalized learning experience, leading to increased motivation and enthusiasm for further language learning tasks. With AI-powered platforms like Duolingo, learners can access language practice anytime and anywhere, empowering them to take control of their learning process and fostering a sense of autonomy and self-regulation.

EFL teachers can also benefit from integrating AI-assisted language learning tools into their instructional strategies. These platforms provide valuable data and insights into each student’s progress and areas of improvement, allowing teachers to tailor their instruction more effectively. By identifying specific language learning challenges, teachers can offer targeted support and guidance to individual students, leading to more efficient and effective language learning outcomes. The AI-supported instruction enables educators to differentiate their teaching approaches, catering to the diverse needs and abilities of their learners.

The study further highlights the potential of AI-powered language learning tools to revolutionize EFL classrooms by creating a personalized and adaptive learning environment. Learners engaging with interactive AI platforms receive instant feedback, comments, and alternative sentences, fostering continuous improvement and instilling confidence in their language abilities. This real-time support creates a supportive and encouraging atmosphere, promoting a positive learning experience. In addition, the integration of AI in language learning opens up exciting avenues for research and development in language pedagogy. As AI technology advances, educators and researchers can explore innovative ways to leverage its potential for improving language learning outcomes and instructional practices. Future studies may delve into the long-term effects of AI-assisted language learning, its applications in different language contexts, and the development of more sophisticated AI tools tailored to specific language learning needs.

## 6. Conclusion

The current study, following Vygotsky’s social constructivism as its theoretical underpinning, examined the role of the AI-powered language learning instruction on Chinese EFL learners’ English learning achievement, L2 motivation, and self-regulated learning. It was found that the AI-assisted language learning tool had a significant impact on the EFL learners’ English learning achievement, L2 motivation, and self-regulated learning on the one hand and that the AI learners outperformed their non-AI counterparts in all the measures. The EFL learners were found to have positive perceptions toward the application of the AI-powered language learning tool in developing their English learning achievement, L2 motivation, and self-regulated learning. In general, the findings might be related to the effectiveness and efficiency of the AI tool in providing the EFL learners with immediate feedback and helping them personalize their language learning environment to be further engaged in the language learning activities.

Although this study has contributed valuable insights into the integration of AI in language instruction and its impact on English learning achievement, L2 motivation, and self-regulated learning, it is imperative to acknowledge several inherent limitations that should be taken into account when interpreting the results. Firstly, it is important to recognize that the research was conducted within a controlled educational setting, specifically focusing on a select group of EFL learners from a single university. Consequently, caution must be exercised when attempting to generalize the findings to broader language learning contexts and more diverse learner populations. In order to enhance the external validity of the outcomes, future research endeavors should strive to encompass a more varied and representative sample of EFL learners. By doing so, the robustness and applicability of the results could be confirmed and extended across various educational environments.

Furthermore, the scope of this study primarily centered around the short-term effects of AI-mediated instruction, dictated by the constraints of the limited timeframe in which the research was conducted. To attain a more comprehensive and holistic understanding of the phenomenon, there is a clear imperative for the implementation of long-term follow-up studies. These extended studies would facilitate an assessment of the sustainability of the observed enhancements in language learning achievement, motivation, and self-regulated learning. The insights garnered from such investigations would be invaluable in illuminating the long-term efficacy of AI-powered language instruction and elucidating its potential implications for long-lasting language learning outcomes.

Additionally, it is noteworthy that the mixed-methods approach employed in this study, while yielding valuable insights into learners’ experiences with AI-mediated instruction, was accompanied by a limitation related to the size of the qualitative sample. The qualitative phase of the study, involving a relatively small number of participants, presents an opportunity for improvement. A more comprehensive and nuanced understanding could be achieved by expanding the qualitative data collection process to encompass a larger and more diversified group of participants. This expansion would undoubtedly enrich the spectrum of perspectives and experiences related to the utilization of AI tools in language learning. The inclusion of a broader range of voices would provide researchers with an enhanced panorama of insights, ultimately contributing to a more robust and exhaustive analysis.

## Data availability statement

The raw data supporting the conclusions of this article will be made available by the authors, without undue reservation. Requests to access these datasets should be directed to LW, Email: Willing5216@163.com.

## Ethics statement

The studies involving humans were approved by College of Foreign Languages, Chongqing College of Mobile Communication, Chongqing, China. The studies were conducted in accordance with the local legislation and institutional requirements. The participants provided their written informed consent to participate in this study.

## Author contributions

LW: Conceptualization, Data curation, Formal analysis, Funding acquisition, Investigation, Methodology, Project administration, Resources, Software, Supervision, Validation, Visualization, Writing – original draft, Writing – review & editing.
